# Sex-Specific Cut-Offs of Seven Adiposity Indicators and Their Performance in Predicting Metabolic Syndrome in Arab Adults

**DOI:** 10.3390/jcm12237280

**Published:** 2023-11-24

**Authors:** Hanan A. Alfawaz, Nasiruddin Khan, Mohammed G. A. Ansari, Malak N. K. Khattak, Gamal M. Saadawy, Nasser M. Al-Daghri

**Affiliations:** 1 Department of Food Science & Nutrition, College of Food & Agriculture Science, King Saud University, Riyadh 11495, Saudi Arabia; 2 Department of Food Science and Human Nutrition, College of Applied and Health Sciences, A’Sharqiyah University, Ibra 400, Oman; 3 Chair for Biomarkers of Chronic Diseases, Biochemistry Department, King Saud University, Riyadh 11451, Saudi Arabiamkhattak@ksu.edu.sa (M.N.K.K.); gsedawy@ksu.edu.sa (G.M.S.)

**Keywords:** obesity, metabolic syndrome components, BMI, Saudi Arabia

## Abstract

This study aimed to assess several indicators of adiposity and their effectiveness in predicting metabolic syndrome (MetS) and identify their cut-off values among general Saudi adults. Consequently, 833 participants (49% male and 51% female) aged 42.2 ± 11.9 years (408 MetS and 425 as controls) were enrolled into this cross-sectional study. Information on demographics, anthropometrics and biochemical results was retrieved from a registry. MetS was defined according to the National Cholesterol Education Program’s (NCEP III) criteria. Overall, the lipid accumulation product (LAP) and waist–TG index (WTI) had the highest discriminatory ability for MetS (area under the curve (AUC): 0.857 and 0.831), respectively, followed by the visceral adiposity index (VAI) and dysfunctional adiposity index (DAI) (AUC: 0.819 and 0.804), respectively. Based on gender, the LAP and WTI were the best indicators for discriminating MetS and presented the highest Youden index values, with cut-off values of 49.8 (sensitivity 68.5%, specificity 82.4%), and 8.7 (sensitivity 70.7%, specificity 81.9%), respectively, in females and 46.2 (sensitivity 85.6%, specificity 76.3%) and 8.9 (sensitivity 73.9%, specificity 84.8%), respectively, in males. The LAP and WTI performed well in both genders with a superior ability to identify MetS in males and could be used to predict MetS in Saudi adults.

## 1. Introduction

Metabolic syndrome (MetS) is a complex, multifaceted health condition that significantly affects global public health. It is a risk factor and a major cause of cardiovascular diseases (CVDs), type 2 diabetes mellitus (T2DM) and all-cause mortality. The criteria of diagnosis for MetS are usually based on the assessment of six indices, including waist circumference (WC), fasting blood glucose (FBG) levels, triglyceride levels (TG), high-density lipoprotein (HDL-C) levels, cholesterol levels and blood pressure (BP) [1].

The prevalence of MetS is at its highest all over the world, with a prevalence of 37.1% in the United States [2], 16.0% in Africa, 21.3% in Asia and 10.5% in Europe [3]. A significant gender difference was also observed in MetS prevalence in several studies from countries such as the US and Europe [4,5]. A recent study in China showed the prevalence of MetS as 33.3% for all participants (46.3% in males and 23.3% in females) [6]. Saudi Arabia is witnessing an alarming rise in obesity and overweight among its general population, including all ages and genders. The most attributable reasons are unhealthy eating behavior due to westernization, a sedentary lifestyle and weight gain [7]. Based on the World Health Organization (WHO) report, the overall prevalence of obesity and overweight in Saudi Arabia is estimated to be 33.7% and 68.2%, respectively. In addition, around 66–75% of adults and 25–40% of children were reported to be either overweight or obese in the Arab region [8]. Studies show a high prevalence of MetS in Saudi Arabia (39.8%: 34.4% in males and 29.2% in females) and 31.6% (45.0% in males and 35.4% in females), according to the NCEP ATP III and International Diabetes Federation (IDF) criteria, respectively, associated with components such low levels of HDL-C, followed by abdominal obesity [9]. The role of adipose tissue dysfunction in the pathogenesis of MetS leading to insulin resistance, oxidative stress and inflammation has been addressed in recent years. Specifically, CVDs were found to be associated with visceral and peripheral adipocytes, the stimulation of the renin–angiotensin–aldosterone system and sex hormones [10,11]. MetS was reported to be highly prevalent among Saudi young people and adults with T2DM [12,13]. In addition, the possible cardiac risk was shown among MetS males and females, with an increased incidence in the elderly and overweight Saudi population [14]. The epidemiological analysis in the Africa Middle East Cardiovascular Epidemiological (ACE) study demonstrated a high prevalence of modifiable cardiovascular risk factors (including MetS components) among Saudi Arabian nationals and expatriates [15]. The high economic burdens attributable to CVDs and diabetes in Saudi Arabia are around $3.5 billion [16] and $0.87 billion, respectively [17].

The ease of early identification and intervention for at-risk individuals may help control the severity of MetS and prevent its further chronic effects. This will significantly reduce the economic burden on society and families, along with an array of benefits to human health. Given the above reasons, the present study chose low-cost, easy-to-calculate, non-invasive and accessible anthropometric indicators to predict MetS.

It has been demonstrated that adiposity measurements may prove more helpful in assessing and predicting the risk of developing MetS [18]. Recently introduced by Amato et al. [19] and Kahn et al. [20], the VAI and LAP are strong and useful indicators for measuring visceral obesity and body fat distribution. Several studies have been performed among various populations, including Korean, Indian and Bangladeshi populations, demonstrating that the LAP and VAI are effective markers for different obesity phenotypes and strong predictors of MetS [21,22,23]. Like the VAI, the DAI was also developed as a surrogate marker of dysfunctional adiposity [24]. In addition, studies show some other simple and inexpensive anthropometric measures used as predictors of MetS, including a body shape index (ABSI) [25], body adiposity index (BAI) [26] and body roundness index (BRI) [27]. Moreover, the WTI has been shown as a valid and superior biomarker of MetS in African Americans [28]. Based on the above evidence, it seems that the use of different adiposity indices is promising to help screen people at high risk of MetS.

Until now, few relevant studies in Saudi Arabia have compared adiposity indices as predictors of MetS. One such recent study demonstrated gender-based cut-off values for WC, waist-hip ratio (WHR) and body mass index (BMI) to identify risk of MetS in the Saudi population [29]. In contrast, another study investigated the predictive ability and association between various anthropometric indices (such as neck circumference, WC, WHR, WC:height and non-anthropometric components of MetS) in non-diabetic Saudi adults [30]. Other studies considered ABSI in demonstrating their relations as predictors for cardiometabolic risk factors in Saudi diabetic patients [31] or the WC to propose cut-off values in predicting increased hypertension or T2DM among Saudi adults [32]. Since no previous studies have looked at such a wide range of adiposity indices, including LAP, VAI, DAI, ABSI, BRI, WTI and BAI, as risk predictors for MetS, therefore, the main aim of this study was to evaluate the gender-based association and effectiveness of different indices in predicting MetS and to determine their cut-off points to identify risk of MetS among the Saudi adult population.

## 2. Materials and Methods

### 2.1. The Study Design and Participants

A total of 833 participants (49% male and 51% female) aged 42.2 ± 11.9 years (408 Mets and 425 without MetS as control subjects) were randomly selected from the master database of the Chair for Biomarkers of Chronic Diseases (CBCD) at King Saud University (KSU, Riyadh, KSA). In brief, this database contains clinical information, blood and serum samples of more than 10,000 Saudi participants (Riyadh Cohort) [33] aged 1–65 years recruited from various primary healthcare clinics (PHCCs) in Riyadh, the Kingdom of Saudi Arabia (KSA), for capital-wide epidemiologic studies on chronic diseases, which was carried out in cooperation with the CBCD and the Ministry of Health (MoH). Participants were asked to fill out a generalized questionnaire containing information on demographics and present/past medical history. Ethical approval was obtained from the Ethics Committee of the College of Science, KSU (No. 8/25/454239, approved 19 April 2013).

### 2.2. Exclusion Criteria

Non-Saudis and those outside the age range were excluded. In addition, subjects with debilitating acute and chronic conditions (e.g., Addison’s disease, cystic fibrosis, Graves’ disease, irritable bowel syndrome, chronic kidney disease, etc.) and those with known genetic syndromes, including syndromic obesity, were excluded. Moreover, pregnant women, subjects with acute infection, acute cardiovascular/cerebrovascular diseases and severely impaired liver/renal function were also excluded from this study.

### 2.3. Sample Collection and Anthropometric and Biochemical Evaluations

The participant data, including anthropometric (BMI, WHR and BP) and biochemical results (glucose and lipid profiles), were meticulously retrieved from the registry. Furthermore, demographic information, including age, sex and medical history, were noted. The BMI, expressed as kg/m^2^, was calculated. All fasting blood samples were assessed and stored in the CBCD (KSU, Riyadh, KSA). The FBG, total cholesterol, HDL-C, TG, calcium, and albumin were measured routinely using a biochemistry analyzer (Konelab 20XT, Thermo Scientific, Vantaa, Finland) [34].

### 2.4. MetS Component and Index Determination

Screening for MetS was conducted using the NCEP III criteria [35] [≥3 out of 5 MetS components, namely, elevated WC, elevated BP, elevated FBG, elevated TG and low HDL-C were categorized as criteria for MetS as applied in our previous study [36]].

1. WC (Central obesity) of >101.6 cm in males and >88.9 cm in females.
2. FBG (Hyperglycemia) > 5.6 mmol/L.
3. Low HDL-C; for males, <1.03 mmol/L, and for females, <1.30 mmol/L.
4. Fasting TG (Hypertriglyceridemia) >1.7 mmol/L.
5. Hypertension; diastolic BP > 85 mmHg and/or systolic BP > 130 mmHg.
  Adiposity index calculations:
  (a) The LAP index [20] was computed based on the following equations among males and females, respectively:
    $$In\;Males,LAP=\left\{WC\left(cm\right)-65\right\}*TG(\frac{mmol}{l})$$
    $$In\;Females,LAP=\left\{WC\left(cm\right)-58\right\}*TG(\frac{mmol}{l})$$
  (b) VAI [19]
    $$In\;Males,VAI=\frac{WC\left(cm\right)}{39.68}+\left(1.88*BMI\right)*\left(\frac{TG}{1.03}\right)*\left(\frac{1.31}{HDL}\right)$$
    $$In\;Females,VAI=\frac{WC\left(cm\right)}{36.58}+\left(1.89*BMI\right)*\left(\frac{TG}{0.81}\right)*\left(\frac{1.52}{HDL}\right)$$
  (c) WTI [37] consisting of WC (cm) and TG (mg/dL). Therefore, the WTI was WTI = $Ln(TG\left(\frac{mg}{dl}\right)*\frac{WC\left(cm\right)}{2})$
  (d) DAI [24]
    $$DAI\;in\;Males=\left(\frac{WC}{22.79+\left(2.68*BMI\right)}\right)*\left(\frac{TG}{1.37}\right)*\left(\frac{1.19}{HDL}\right)$$
    $$DAI\;in\;Females=\left(\frac{WC}{24.02+\left(2.38*BMI\right)}\right)*\left(\frac{TG}{1.32}\right)*\left(\frac{1.43}{HDL}\right)$$
  (e) BRI: [38]
    $$BRI\;for\;Both\;Genders=364.2-365.5*(1-\frac{\left({\left(\frac{WC}{2\pi }\right)}^{2}\right)}{{\left(0.5*{\left(height\right)}^{2}\right)}^{0.5}})$$
  (f) ABSI: A body shape index (ABSI) was calculated using the Krakauer and Krakauer equation [25]:
    $$ABSI={WC/((BMI)}^{2/3}*{\left(height\right)}^{1/2}$$
  (g) BAI: The BAI was calculated as proposed by Bergman et al. [39]:
    $$BAI=\frac{Hip}{Height\sqrt[{}]{Height}}-18$$

### 2.5. Data Analysis

SPSS version 28.0 (SPSS, Inc., Chicago, IL, USA) was used to analyze the data. We used the Kolmogorov–Smirnov test to ensure that our data were normally distributed. Normally distributed data were presented as means and standard deviation (SD). Non-normal data were presented as medians (25th and 75th percentiles). Non-Gaussian variables were log-transformed prior to parametric analysis. Categorical variables were shown as frequencies (percentages) and analyzed using a chi-square test. Student-independent t- and Mann–Whitney U tests were performed to check the mean and median difference between controls vs. MetS. Further, one-way ANOVA was performed for metabolic scores for different indices. Multinomial logistic regression analysis was performed for MetS to identify the odds ratio (95%CI) with and without adjustment for different indices. ROC analysis was performed to determine the cut-off for MetS on sensitivity, specificity and Youden index J. Statistical significance was defined as *p* < 0.05.

## 3. Results

### 3.1. Clinical Characteristics of the Subjects

Table 1 represents the clinical characteristics among the control (N = 425) and MetS (408) subjects. All adiposity index (LAP, VAI, DAI, ABSI, BRI, WTI and BAI) values were significantly higher (*p* < 0.001) among the MetS subjects than the control.

The gender-based, clinical and anthropometric characteristics of the MetS participants are shown in Table 2. Variables such as age, weight, BMI, waist measurement, hip measurement, WHR and Waist-to-Height Ratio (WHtR) were significantly higher (all variables *p* < 0.001) in MetS females than in the control. On the other hand, the male MetS subjects showed a significantly higher weight, BMI, waist measure, hip measure, WHR and WHtR (all variables *p* < 0.001) than the controls. Moreover, BP, cholesterol profile, TG, glucose levels and indices such as the LAP, VAI, DAI, WTI, BAI, ABSI and BRI were significantly higher among both the male and female MetS groups than control (Table 2).

### 3.2. Prevalence of MetS and Its Components among Saudi Adults

**Figure 1:**
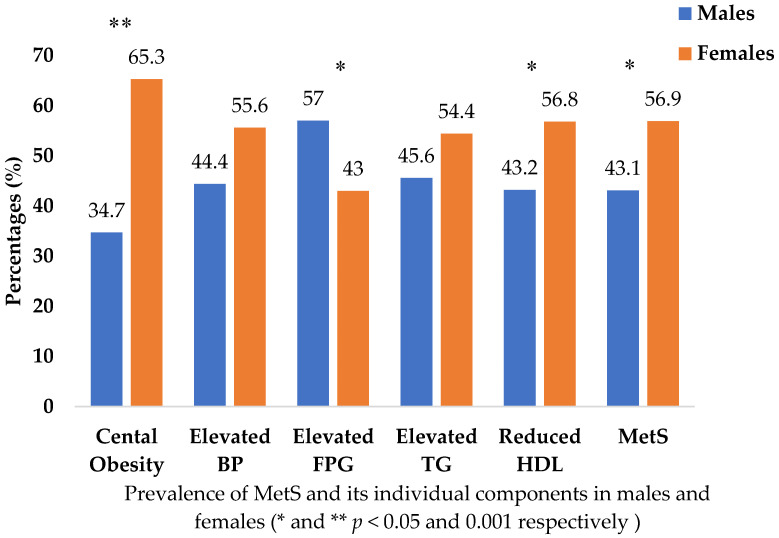
Gender-based prevalence of MetS and its components.

Figure 1 depicts the gender-based prevalence of MetS among subjects. No significant difference exists in the proportion of males or females with MetS in the selected population sample. In terms of the prevalence of MetS components, females had significantly higher values of central obesity (65.3%, *p* < 0.001), and reduced HDL-C (56.8%, *p* < 0.05) compared to males. The other MetS components, such as elevated BP (55.6%) and elevated TG (54.4%), were non-significantly higher in females than males. The males exhibited a significantly higher percentage only for elevated FPG (57%, *p* < 0.05) than females (43%).

### 3.3. Association of Adiposity Indices as Predictors of MetS

After adjusting (age, BMI and gender) among the overall participants, and for BMI in males and age and BMI in females, multiple logistic regression analyses demonstrated that the odds of having MetS were strongly associated with the LAP and WTI in the overall participants (OR = 33.47, 95%CI: 19.43–57.65; and OR = 27.98, 95%CI:16.85–45.48), respectively, as well as among both males and females (OR =34.81, 95%CI: 15.89–76.20 and OR = 49.78, 95%CI: 19.44–127.48; OR = 28.75, 95%CI: 13.58–60.84 and OR = 22.29, 95%CI: 10.82–45.89), respectively, which increased several-fold with increasing tertile (*p* < 0.001). For the male participants, the LAP and WTI had the highest odds ratio in predicting MetS, at odds with for the results for females. The DAI and VAI showed a lower odds ratio as predictors for metabolic risk factors than the LAP and WTI among males and females. Moreover, ABSI and BAI were among the weakest determinants for MetS risk among all participants and for both genders (Table 3).

### 3.4. ROC Curve of Different Adiposity Indices as a MetS Indicator

**Figure 2:**
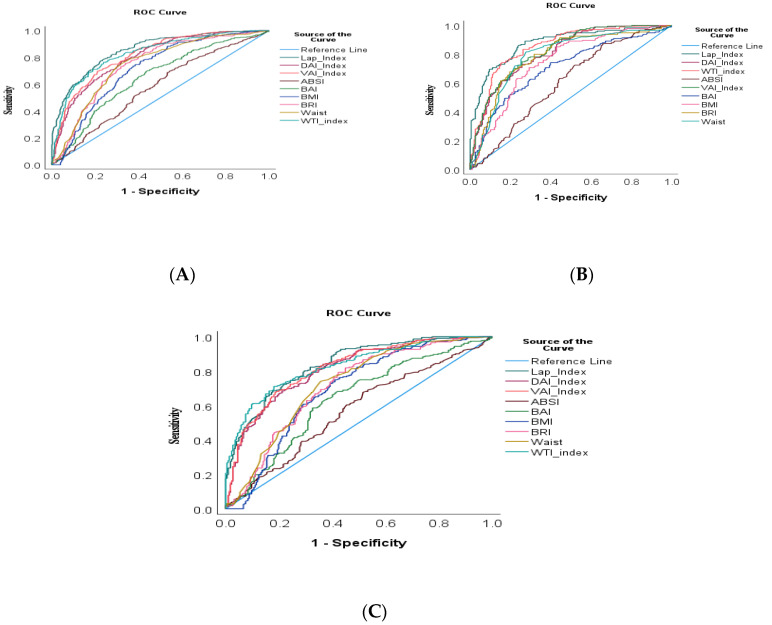
ROC curve of different anthropometric indicators for predicting MetS in (**A**) Overall participants, (**B**) male and (**C**) female.

ROC analysis was performed (Figure 2) to illustrate the discriminating ability of different adiposity indicators for MetS in all participants and compared between both males and females. In the overall sample, an excellent ability to discriminate MetS was shown by the LAP (all participants; AUC: 0.857; 95%CI: 0.831–0.880), followed by the WTI (AUC: 0.831; 95%CI: 0.804–0.856), VAI (AUC: 0.819; 95%CI: 0.791–0.845) and DAI (AUC: 0.804; 95%CI: 0.775–0.830), respectively. Indices such as the BRI and waist measure exhibited almost the same (AUC: 0.746, 0.747, respectively) values. The BMI and BAI showed lower discriminating values for MetS (AUC: 0.71, 0.64, respectively), while ABSI had the most insufficient discrimination power for MetS (AUC: 0.557; 95%CI: 0.543–0.611) (Table 4).

In male, the LAP, WTI, VAI and DAI had larger AUCs—AUC: 0.877, 95%CI: (0.840–0.909); AUC: 0.856, 95%CI: (0.816–0.890); AUC: 0.825, 95%CI: (0.783–0.862); and (AUC: 0.816, 95%CI: (0.773–0.854), respectively—than other indices. In females, the same indices, namely the LAP and WTI, had larger AUCs—AUC: 0.840, 95%CI: (0.803–0.872) and (AUC: 0.831, 95%CI: (0.793–0.864), respectively—while the VAI and DAI areas (AUC: 0.818, 95%CI: (0.780–0.852) and AUC: 0.811, 95%CI: (0.772–0.845)) were same as in males. Moreover, the areas under the curve for the LAP and WTI were more significant for males than females (AUC: 0.877 vs. 0.840 and 0.856 vs. 0.831), respectively. In addition, the areas under the curve for other indices such as BMI, waist measure, BAI and BRI were also higher in males than females.

The optimal cut-off values, Youden’s index and the respective sensitivity and specificity values of the indicators are presented in Table 4. In the overall sample, the LAP and WTI showed the highest Youden’s index values, identifying a cut-off value of 46.74 (sensitivity 77.59% and specificity 77.41%) for the LAP and 8.71 (sensitivity 75.25% and specificity 76.94%) for the WTI. The cut-off values for the VAI and DAI were 2.67 (sensitivity 69.95% and specificity 78.59%) and 3.28 (sensitivity 76.96 and specificity 67.76), respectively. The indices such as the BRI and waist measure exhibited the cut-off values 4.78 (sensitivity 75.49% and specificity 66.12%) and 92.2 (sensitivity 75.98% and specificity 67.53%), respectively. The BMI and BAI showed cut-off values of 28.26 and 31.63, respectively, while the ABSI cut-off value was 0.079.

Among males, the LAP index presented the highest Youden’s index values with cut-off values of 46.2 (sensitivity 85.63%, specificity 76.26%), followed by 8.92 (sensitivity 73.86%, specificity 84.85%) for the WTI, respectively, as a predictor of MetS. Similarly, the LAP and WTI presented the highest Youden’s index values to predict MetS in females, with cut-off values of 49.82 (sensitivity 68.53%, specificity 82.38%) for the LAP, and 8.68 (sensitivity 70.69%, specificity 81.94%) for the WTI, respectively.

Table 5 represents the number and mean value of different metabolic scores and adiposity indices. Among all participants, the mean value of all adiposity indices showed a pattern of increase with an increase in MetS components or increased metabolic abnormality.

## 4. Discussion

The escalating prevalence of obesity and MetS in the Saudi population has raised significant concerns about the associated health risks and urges effective early detection and screening methods. The use of various adiposity indicators may prove an effective and easily applicable tool to attain this goal. Thus, we aimed to evaluate the effectiveness of different adiposity indices as predictors for MetS and determine their cut-off values. Our results showed that the prevalence of MetS was high in the adult population, specifically among females. At the same time, the LAP and WTI as adiposity indices were better predictors for MetS, followed by the DAI and VAI.

According to the NCEP ATP III and IDF criteria, a study performed by Al-Rubeaan and colleagues [29] showed a lower prevalence of MetS in Saudi females than in males (29.2 vs. 34.4%) and (35.4% vs. 45.0%), respectively. However, a higher prevalence of MetS was demonstrated in Qatari female and male participants (42.5 vs. 56.7%), respectively [40]. Our present study was inconsistent with these findings [29,40], showing a higher prevalence of MetS in females than males (56.9 vs. 43.1%), respectively. The possible reason for this discrepancy could be explained by differences in body fat distribution and daily lifestyle behavior among both genders. A study performed among the adult population living in Sharjah and the Northern Emirates showed a higher prevalence of MetS (NCEP/ATP III guidelines) in females (38.7%) than in males (28.8%) [41]. Although, based on gender, the prevalence of MetS components was much higher, our present study partially supports this finding [41], showing a higher prevalence of MetS among females than males. The present study demonstrated higher metabolic abnormalities among females, including central obesity and reduced HDL-C, than in males. In contrast, males exhibited a higher incidence of elevated FPG than females. The results obtained from the United Arab Emirates National Diabetes and Lifestyle Study (UAEDIAB) [41] demonstrated a significantly higher prevalence of increased WC and lower HDL-C in females than men, 67 vs. 53.7% and 44.1 vs. 36.3%, respectively. Our present study corroborated this finding, demonstrating higher central obesity and reduced HDL-C in females than males (65.3 vs. 34.7%) and (56.8 vs. 43.2%), respectively. High FPG, BP and TG levels were demonstrated among Jordanian [42] and Arab and Asian males [43]. Our present findings partially support these results, with high FPG levels in males, but do not support the presence of other metabolic components. Similarly, the Saudi community showed a high prevalence of metabolic abnormalities like elevated TG, BP and FPG in males, with low HDL-C levels more common in females [29].

A recent study among the Turkish adult population [44] demonstrated the LAP index with a cut-off value 44.5 (AUC = 0.915) as a strong predictive tool for the early detection of MetS. In addition, a Brazilian study among 141 HIV-infected patients on antiretroviral therapy showed the LAP index as a tool for diagnostic screening for MetS with a best cut-off value of 59.4 (sensitivity 80%, specificity 79%) and an AUC = 0.875. Based on gender, the ROC curve of the same study showed the LAP index as the best indicator for MetS with cut-off values of 56.3 (sensitivity 100%, specificity 82% and AUC = 0.929) and 52.0 (sensitivity 78%, specificity 74% and AUC = 0.838), among males and females, respectively [45]. A study performed by Rajendran and colleagues [46] among Indian young adults demonstrated the LAP index with the highest area under the curve (0.882 and 0.905 in male and female subjects, respectively), with the cut-off value for the LAP being 45.65 in males (sensitivity and specificity of 80%) and 46.91 in females (sensitivity and specificity of 88%). Our present study supports the above findings with slight deviation, demonstrating the excellent ability of the LAP index in predicting the risk of MetS with a cut-off value of 46.74 (sensitivity 77.59% and specificity 77.41%, AUC = 0.857) among all participants, which is higher than the Turkish (44.5) but lower than the Brazilian (59.4) LAP index cut off value. Moreover, based on gender, our present study corroborates these findings, showing LAP index cut-off values of 46.2 (sensitivity 85.63%, specificity 76.26%) and 49.82 (sensitivity 68.53%, specificity 82.38%) for males and females, respectively, which is closer to the LAP index cut-off values shown by Rajendran et al. (45.65 in males and 46.91 in females) [46].

A cross-sectional study among non-diabetic adults [37] demonstrated the WTI as a reliable and inexpensive tool to screen MetS specifically in females rather than males. The cut-off values for the WTI were 8.7 for females and 8.9 for males, respectively. Our present study corroborates the above findings, showing similar cut-off values for the WTI of 8.68 (sensitivity 70.69%, specificity 81.94%) and 8.92 (sensitivity 73.86%, specificity 84.85%) for females and males, respectively. A recent study among South Korean adults [21] demonstrated the VAI as an effective index for predicting the metabolically unhealthy obesity phenotype. For the VAI, the AUCs were 0.877, 0.849, and 0.921 for all participants, men and women, respectively. The optimal VAI cut-off values were 1.83 in men and 1.58 in women. Another study [43] showed the AUC and cut-off value for the VAI as 0.836 and 1.61, respectively, for all participants. Moreover, the same study demonstrated the DAI as having the highest discriminatory ability for MetS (AUC = 0.921) and ABSI as having the lowest discrimination power for MetS (AUC = 0.606). The cut-off value for the DAI in predicting MetS was 0.9799, while for ABSI, the value was 0.075.

Our present study showed the VAI and DAI as having a discriminating ability for MetS but as being less effective than the LAP and WTI. However, our current study supports the above findings by exhibiting ABSI as the lowest discriminator for MetS among Saudi adults. There are contrasting results related to the BRI and BAI. A study among Chinese postmenopausal women showed that neither the BAI nor BRI was superior to traditional obesity indices in predicting MetS. Moreover, the BAI showed the weakest predictive ability [47]. In contrast, a study among Southern Indian adults demonstrated the BRI as a strong predictor of MetS [48]. The above finding is consistent with our present study showing the BAI and BRI as weak predictors of MetS, with the BAI as the weakest among the Saudi population.

### Strengths and Limitations

There are several strengths of the current study. First, the sample size of 833 multi-central participants properly represents a population. Secondly, to the best of our knowledge, this is the first study to evaluate the gender-based effectiveness of seven adiposity indices in predicting MetS and to determine their cut-off points risk among the Saudi adult population. The authors, however, acknowledge certain limitations. The results of the current study might be limited due to its cross-sectional nature in comparing the ability of anthropometric indices to predict the components of metabolic abnormality. Thus, causality cannot be clearly determined. In addition, the possibility of excessive ROC curve extrapolation could be detrimental. Although menopausal status could be identified in this study, the present data lack age-group-based analysis, which may have limited the assessment of the performance of each indicator with advancing age.

## 5. Conclusions

The present study evaluated the effectiveness of various adiposity indicators for determining MetS. In addition, the results showed a higher prevalence of MetS components in Saudi females than males. The anthropometric indicators LAP and WTI were better than the other indices in predicting MetS in both genders, followed by the DAI and VAI. However, these indicators (LAP and WTI) were better than the other indices in predicting MetS among males than females. ABSI and the BAI exhibited weak performance as determinants for MetS risk among all participants.

The results of the current study might help select a better and more affordable index for MetS prediction and screening. The findings advocate for their continuous use to ensure better prospects for detecting and treating MetS in Saudi adults. Although these adiposity indicators show a certain predictive power for MetS, there is a need for future clinical studies that allow progress in defining the cut-offs in MetS prevention. In addition, future studies encompassing other contributing factors to MetS, such as dietary and lifestyle behaviors, can be studied in relation to the anthropometric indices.

## Figures and Tables

**Table 1:** Clinical characteristics of the subjects.

Parameters	Overall Subject		Effect Size	*p*-Value	MetS and Gender Interaction *p*-Value	Multiple Comparison *p*-Value for Seven Parameters
	Control	MetS				
N (M/F)	425 (198/227)	408 (176/232)				
Age	39.7 ± 13.8	44.8 ± 9.1	0.079	<0.001	0.02	
Height (cm)	161.6 ± 9.2	160.8 ± 8.5	0.426	0.23	0.001	
Weight (Kg)	71.4 ± 14.8	80.7 ± 12.2	0.128	<0.001	0.001	
BMI (kg/m^2^)	27.5 ± 5.9	31.3 ± 4.4	0.169	<0.001	0.001	
Waist (cm)	89.0 ± 12.8	100.2 ± 12.8	0.175	<0.001	0.003	
Hip (cm)	101.1 ± 13.7	108.6 ± 14.4	0.117	<0.001	0.002	
WHR	0.89 ± 0.12	0.93 ± 0.15	0.129	<0.001	<0.001	
WHtR	0.55 ± 0.09	0.62 ± 0.08	0.174	<0.001	0.002	
Systolic BP	114.5 ± 10.8	125.6 ± 14.3	0.171	<0.001	0.004	
Diastolic BP	74.5 ± 7.2	79.6 ± 8.4	0.10	<0.001	0.001	
FBG (mmol/L)	5.47 ± 1.6	7.18 ± 1.9	0.185	<0.001	0.001	
Chol (mmol/L)	4.94 ± 1.1	5.26 ± 1.1	0.027	<0.001	0.007	
HDL-C (mmol/L)	1.07 ± 0.4	0.86 ± 0.28	0.177	<0.001	<0.001	
LDL-C (mmol/L)	3.29 ± 0.9	3.42 ± 1.0	0.006	0.07	0.002	
TG (mmol/L)	1.16 (0.9–1.46)	1.86 (1.37–2.56)	0.222	<0.001	0.009	
Atherogenic Index (AI)	3.07 (2.2–4.26)	3.89 (2.97–5.03)	0.069	<0.001	0.103	0.006
CRI	1.12 (0.76–1.69)	2.13 (1.46–3.45)	0.204	<0.001	<0.001	<0.001
LAP Index	30.03 (19.3–45.82)	70.96 (47.81–101.2)	0.294	<0.001	0.02	<0.001
VAI	1.78 (1.23–2.55)	3.60 (2.39–5.47)	0.173	<0.001	0.008	<0.001
DAI	2.49 (1.73–3.69)	4.93 (3.37–7.59)	0.183	<0.001	0.02	<0.001
WTI	8.43 ± 0.43	9.05 ± 0.51	0.362	<0.001	0.006	<0.001
BAI	31.63 ± 8.6	35.59 ± 8.6	0.287	<0.001	0.002	<0.001
ABSI	0.077 ± 0.008	0.079 ± 0.009	0.073	<0.001	0.001	<0.001
BRI	4.54 ± 1.9	6.12 ± 2.10	0.154	<0.001	0.002	<0.001

Note: Data presented as mean ± SD and median (1st–3rd) percentiles for Gaussian and non-Gaussian variables. *p*-value was significant at the 0.05 and 0.01 levels.

**Table 2:** Gender-based clinical characteristics of the subjects.

Parameters	Males		*p*-Value	Females		*p*-Value
	Control	MetS		Control	MetS	
N	198	176		227	232	
Age	42.9 ± 15.4	44.7 ± 9.5	0.197	36.8 ± 11.6	44.9 ± 8.7	<0.001
Height (cm)	168.0 ± 6.7	167.2 ± 6.6	0.250	155.99 ± 7.2	156.0 ± 6.4	0.940
Weight (Kg)	73.2 ± 14.1	83.7 ± 12.4	<0.001	69.8 ± 15.3	78.5 ± 11.6	<0.001
BMI (kg/m^2^)	25.9 ± 4.9	29.9 ± 4.2	<0.001	28.8 ± 6.5	32.2 ± 4.3	<0.001
Waist (cm)	89.9 ± 12.1	102.8 ± 12.3	<0.001	88.3 ± 13.4	98.3 ± 12.8	<0.001
Hip (cm)	97.0 ± 12.6	105.7 ± 14.4	<0.001	104.7 ± 13.7	110.8 ± 14.1	<0.001
WHR	0.94 ± 0.14	0.99 ± 0.17	0.001	0.85 ± 0.09	0.89 ± 0.13	<0.001
WHtR	0.54 ± 0.08	0.62 ± 0.07	<0.001	0.57 ± 0.07	0.63 ± 0.08	<0.001
Systolic BP	116.7 ± 10.5	16.1 ± 12.9	<0.001	112.7 ± 10.7	125.3 ± 15.3	<0.001
Diastolic BP	75.2 ± 6.9	79.7 ± 7.7	<0.001	73.9 ± 7.3	79.6 ± 8.8	<0.001
FBG (mmol/L)	5.63 ± 1.7	7.20 ± 2.2	<0.001	5.33 ± 1.6	7.15 ± 2.0	<0.001
Chol (mmol/L)	4.86 ± 1.1	5.39 ± 1.2	<0.001	5.01 ± 1.1	5.16 ± 1.0	0.141
HDL-C (mmol/L)	0.95 ± 0.32	0.75 ± 0.23	<0.001	1.17 ± 0.38	0.94 ± 0.28	<0.001
LDL-C (mmol/L)	3.25 ± 1.1	3.47 ± 1.2	0.051	3.32 ± 0.9	3.37 ± 0.9	0.59
TG (mmol/L)	1.2 (0.9–1.6)	2.1 (1.6–3.1)	<0.001	1.1 (0.87–1.38)	1.7 (1.2–2.22)	<0.001
Atherogenic Index (AI)	3.45 (2.5–4.7)	4.68 (3.38–6.0)	<0.001	2.76 (2.1–33.91)	3.56 (2.84–4.39)	<0.001
CRI	1.41 (0.99–2.03)	3.03 (1.87–4.34)	<0.001	0.92 (0.7–1.35)	1.72 (1.29–2.70)	<0.001
LAP Index	29.9 (19.3–46.1)	78.6 (53.3–121.9)	<0.001	31.0 (19.3–45.2)	63.9 (44.5–93.0)	<0.001
VAI	1.83 (1.26–2.63)	4.03 (2.5–6.5)	<0.001	1.74 (1.22–2.42)	3.49 (2.3–5.14)	<0.001
DAI	2.88 (1.9–4.13)	6.16 (3.8–10.3)	<0.001	2.22 (1.58–3.23)	4.46 (3.0–6.55)	<0.001
WTI	8.54 ± 0.5	9.25 ± 0.5	<0.001	8.33 ± 0.39	8.89 ± 0.44	<0.001
BAI	26.7 ± 6.6	31.0 ± 7.3	<0.001	35.9 ± 7.8	39.0 ± 7.9	<0.001
ABSI	0.079 ± 0.008	0.082 ± 0.007	<0.001	0.075 ± 0.007	0.077 ± 0.009	0.02
BRI	4.17 ± 1.6	5.91 ± 1.9	<0.001	4.87 ± 2.1	6.28 ± 2.2	<0.001

Note: Data presented as mean ± SD and median (1st–3rd) percentiles for Gaussian and non-Gaussian variables. *p*-value was significant at the 0.05 and 0.01 levels.

**Table 3:** Multinomial logistic regression MetS-dependent parameters.

Parameters	Overall		Males		Females	
	Odds Ratio (95%) CI	*p*-Value	Odds Ratio (95%) CI	*p*-Value	Odds Ratio (95%) CI	*p*-Value
Tertile BAI						
T1 (≤29.58)	1		1		1	
T2 (29.58–37.10)	1.4 (0.9–2.1)	0.12	3.3 (2.1–5.3)	<0.001	0.8 (0.4–1.6)	0.56
T3 (>37.10)	1.6 (0.9–2.6)	0.09	5.2 (2.5–10.7)	<0.001	1.2 (1.-6.4)	0.79
Tertile WTI						
T1 (≤8.47)	1		1		1	
T2 (8.48–8.92)	3.4 (2.2–5.2	<0.001	5.6 (2.2–14.2)	<0.001	2.8 (1.7–4.6)	<0.001
T3 (>8.92)	27.9 (16.9–45.5)	<0.001	49.8 (19.4–127.5)	<0.001	22.3 (10.8–45.9)	<0.001
Tertile ABSI						
T1 (≤0.0744)	1		1		1	
T2 (0.0745–0.816)	2.7 (1.8–3.9)	<0.001	4.4 (2.2–8.9)	<0.001	2.0 (1.3–3.3)	0.003
T3 (>0.816)	3.2 (2.1–4.8)	<0.001	6.3 (3.1–12.8)	<0.001	1.9 (1.1–3.4)	0.02
Tertile BRI						
T1 (≤4.17)	1		1		1	
T2 (4.18–6.04)	4.1 (2.7–6.3)	<0.001	4.6 (2.6–8.4)	<0.001	3.4 (1.8–6.1)	<0.001
T3 (>6.04)	4.9 (2.9–8.3)	<0.001	7.5 (3.4–16.6)	<0.001	3.2 (1.6–64)	0.001
Tertile LAP						
T1 (≤33.30)	1		1		1	
T2 (33.31–63.0)	5.8 (3.7–9.3)	<0.001	4.1 (1.9–9.3)	<0.001	7.1 (3.8–13.1)	<0.001
T3 (>63.0)	33.5 (19.4–57.6)	<0.001	34.8 (15.9–76.2)	<0.001	28.8 (13.6–60.8)	<0.001
Tertile DAI						
T1 (≤2.63)	1		1		1	
T2 (2.64–4.63)	6.5 (4.2–10.3)	<0.001	13.0 (5.3–32.1)	<0.001	4.8 (2.8–8.2)	<0.001
T3 (>4.63)	28.9 (17.5–47.8)	<0.001	31.2 (20.1–130.3)	<0.001	26.2 (13.3–51.8)	<0.001
Tertile VAI						
T1 (≤1.92)	1		1		1	
T2 (1.93–3.39)	5.1 (3.3–7.8)	<0.001	5.9 (2.9–12.2)	<0.001	4.5 (2.6–7.8)	<0.001
T3 (>3.39)	23.7 (14.6–38.1)	<0.001	30.4 (14.0–66.4)	<0.001	20.2 (10.7–37.9)	<0.001
Tertile AI						
T1 (≤2.63)	1		1		1	
T2 (2.64–4.63)	2.88 (1.97–4.21)	<0.001	1.81 (0.94–3.48)	0.075	3.93 (2.41–6.40)	<0.001
T3 (>4.63)	3.69 (2.48–5.47)	<0.001	3.98 (2.19–7.23)	<0.001	2.86 (1.65–4.94)	<0.001
Tertile CRI						
T1 (≤1.16)	1		1		1	
T2 (1.17–2.06)	5.12 (3.32–7.88)	<0.001	9.08 (3.14–26.20)	<0.001	4.43 (2.67–7.34)	<0.001
T3 (>2.06)	21.96 (13.4–35.9)	<0.001	45.36 (15.57–132.2)	<0.001	15.53 (8.15–29.61)	<0.001

Note: Adjusted odds ratio (95%) CI presented for overall adjustment (age, BMI, gender), males (BMI) and females (age and BMI).

**Table 4:** RUC, optimal cut-off values, sensitivity, specificity and Youden index for the adiposity indicators and MetS.

Parameters	AUC (95%) CI	Cut-Off	*p*-Value	Sensitivity (%)	Specificity (%)	Youden Index J
Overall						
BMI	0.716 (0.681–0.746)	>28.26	<0.001	74.75	61.65	0.364
Waist	0.747 (0.716–0.776)	>92.2	<0.001	75.98	67.53	0.435
VAI	0.819 (0.791–0.845)	>2.67	<0.001	69.95	78.59	0.485
ABSI	0.557 (0.543–0.611)	>0.079	<0.001	70.10	45.65	0.157
BAI	0.649 (0.615–0.681)	>31.63	<0.001	69.36	56.47	0.258
DAI	0.804 (0.775–0.830)	>3.28	<0.001	76.96	67.76	0.447
BRI	0.746 (0.715–0.775)	>4.78	<0.001	75.49	66.12	0.416
LAP	0.857 (0.831–0.880)	>46.74	<0.001	77.59	77.41	0.550
WTI	0.831 (0.804–0.856)	>8.71	<0.001	75.25	76.94	0.522
AI	0.645 (0.607–682)	>3.31	<0.001	58.32	67.45	0.256
CRI	0.787 (0.757–0.817)	>1.38	<0.001	64.52	79.11	0.435
Males						
BMI	0.749 (0.702–0.792)	>25.88	<0.001	85.23	57.07	0.423
Waist	0.789 (0.744–0.829)	>93.98	<0.001	81.25	72.22	0.535
VAI	0.825 (0.783–0.862)	>2.57	<0.001	74.71	74.24	0.489
ABSI	0.602 (0.551–0.651)	>0.075	<0.001	87.5	33.84	0.213
BAI	0.705(0.656–0.751)	>27.4	<0.001	73.86	59.60	0.335
DAI	0.816 (0.773–0.854)	>4.02	<0.001	73.3	74.24	0.475
BRI	0.787 (0.742–0.827)	>4.38	<0.001	80.11	68.18	0.483
LAP	0.877 (0.840–0.909)	>46.2	<0.001	85.63	76.26	0.619
WTI	0.856 (0.816–0.890)	>8.92	<0.001	73.86	84.85	0.587
AI	0.662 (0.607–0.717)	>4.36	<0.001	71.10	57.23	0.287
CRI	0.811 (0.768–0.854)	>1.91	<0.001	72.70	74.10	0.469
Females						
BMI	0.690 (0.645–0.732)	>29.51	<0.001	75.0	59.03	0.340
Waist	0.721 (0.677–0.761)	>91	<0.001	74.14	64.32	0.384
VAI	0.818 (0.780–0.852)	>2.69	<0.001	67.24	81.50	0.487
ABSI	0.568 (0.521–0.614)	>0.073	0.011	68.97	46.70	0.157
BAI	0.635 (0.589–0.679)	>36.86	<0.001	66.38	59.91	0.263
DAI	0.811 (0.772–0.845)	>3.4	<0.001	68.53	78.85	0.474
BRI	0.710 (0.667–0.752)	>4.78	<0.001	79.31	57.71	0.370
LAP	0.840 (0.803–0.872)	>49.82	<0.001	68.53	82.38	0.509
WTI	0.831 (0.793–0.864)	>8.68	<0.001	70.69	81.94	0.526
AI	0.649 (0.598–0.70)	>2.86	<0.001	53.31	75.14	0.279
CRI	0.831 (0.793–0.864)	1.27	<0.001	72.74	75.90	0.485

**Table 5:** Mean level of different indices for metabolic scores.

Parameters	0	1	2	3	4	5	*p*-Value
N	24	118	283	174	180	54	
Overall							
BMI	22.57 ± 4.3	24.71 ± 4.7	29.03 ± 5.9	30.25 ± 4.7	31.96 ± 3.9	31.14 ± 4.2	<0.001
Waist	80.65 ± 6.6	82.88 ± 10.1	92.33 ± 12.9	98.08 ± 13.8	100.91 ± 11.9	105.01 ± 10.5	<0.001
VAI	1.07 ± 0.4	1.77 ± 1.02	2.47 ± 1.98	3.89 ± 3.10	5.21 ± 4.48	6.46 ± 4.30	<0.001
ABSI	0.0788 ± 0.008	0.0765 ± 0.007	0.077 ± 0.009	0.079 ± 0.009	0.079 ± 0.008	0.081 ± 0.008	0.003
BAI	25.59 ± 4.21	28.81 ± 6.77	33.32 ± 9.1	35.27 ± 9.34	35.43 ± 8.19	37.15 ± 7.42	<0.001
DAI	1.68 ± 0.66	2.57 ± 1.48	3.52 ± 2.9	5.71 ± 5.41	7.27 ± 6.6	8.98 ± 6.1	<0.001
BRI	3.13 ± 0.6	3.64 ± 1.42	5.04 ± 1.95	5.77 ± 2.20	6.27 ± 2.10	6.77 ± 1.52	<0.001
LAP	17.02 ± 8.5	23.49 ± 13.3	41.0 ± 20.7	62.19 ± 30.1	92.41 ± 58.2	118.8 ± 64.6	<0.001
WTI	8.15 ± 0.34	8.23 ± 0.37	8.54 ± 0.42	8.86 ± 0.43	9.13 ± 0.54	9.36 ± 0.38	<0.001
AI	2.43 ± 0.8	3.59 ± 2.2	3.77 ± 2.4	4.56 ± 2.9	4.39 ± 2.4	4.96 ± 2.7	<0.001
CRI	0.82 ± 0.3	1.18 ± 0.7	1.63 ± 1.5	2.39 ± 1.9	3.10 ± 2.5	3.98 ± 2.8	<0.001

Note: Data presented as mean ± SD. *p*-value presented *p* for trend.

## Data Availability

Data will be made available upon request by the corresponding author.
